# Time-Domain Airborne Electromagnetic Inversion with Gradient Guidance and Structural Enhancement

**DOI:** 10.3390/s26165099

**Published:** 2026-08-12

**Authors:** Dajun Li, Yuan Gao, Yaoming Wang, Wei Su, Xingwang Li, Xuanlong Shan

**Affiliations:** 1 College of Surveying and Prospecting Engineering, Jilin Jianzhu University, Changchun 130118, China; gaoyuan0539@student.jlju.edu.cn (Y.G.); wangyaoming@student.jlju.edu.cn (Y.W.); suwei@jlju.edu.cn (W.S.); lixingwang@student.jlju.edu.cn (X.L.); 2 College of Earth Sciences, Jilin University, Changchun 130012, China; shanxl@jlu.edu.cn

**Keywords:** airborne electromagnetics, gradient-based inversion, neural network, physical constraint, data-driven

## Abstract

**Highlights:**

**What are the main findings?**

- A gradient-guided iterative enhancement (GGIE) inversion method for time-domain airborne electromagnetic data is developed, which establishes a closed-loop feedback mechanism between gradient-based inversion and neural network prediction.
- Gauss–Newton (GN) inversion and U-Net provide a gradient guidance and structural enhancement of model convergence, respectively.
- A coarse inverted model (IM) from GN inversion and a predicted model (PM) from U-Net are fused by an adaptive weight coefficient method.

**What are the implications of the main findings?**

- The method is designed to improve the recovery of complex multilayer resistivity structures, especially thin interbedded layers that are easily smoothed by conventional regularized inversion.
- Synthetic and field examples indicate that GGIE provides a more stable compromise between structural resolution and data fitting than standalone GN inversion or neural-network prediction.

**Abstract:**

Gradient-based inversion methods are widely used for time-domain airborne electromagnetic (AEM) data, but their results are commonly affected by the initial model and regularization-induced smoothing. These limitations are particularly evident when thin layers or alternating high- and low-resistivity structures need to be resolved. To address this problem, we propose a gradient-guided iterative enhancement (GGIE) inversion that combines a limited-iteration Gauss–Newton (GN) inversion with a lightweight U-Net. In each GGIE inversion iteration, the GN module first produces a coarse inverted model (IM) that preserves the main data-driven geoelectric trend but is still affected by regularization-induced smoothing. The trained U-Net then predicts a structurally enhanced model (PM) from the observed data and the IM. A data-misfit-guided adaptive approach is proposed to calculate the weight coefficients of the IM and PM and to construct an update model (UM). These coefficients are further smoothed by a momentum term so that the relative contributions of the IM and the PM are adjusted adaptively during the iterations. This design reduces error propagation from either component alone and dynamically balances learned structural enhancement with physics-based data consistency. The UM then serves as the initial model for the subsequent GN inversion. GGIE inversion is tested on synthetic data, and the results show that it is most beneficial for complex multilayer structures, for which it reduces the mean relative error and root mean squared error (RMSE) by 49.0% and 28.6%, respectively. Compared with the physics-informed neural network (PINN) baseline, GGIE inversion reduces the model relative error, log-domain RMSE, and data misfit by 19.4%, 7.0%, and 71.7%, respectively. Moreover, compared with U-Net alone, GGIE inversion reduces the data misfit by 85.4%. The proposed method is further applied to field data acquired from the Fox River area in Wisconsin, USA. The main advantage of GGIE inversion is its ability to resolve complex multilayered structures, thin layers, and sharp resistivity contrasts with improved accuracy and stability.

## 1. Introduction

The time-domain airborne electromagnetic method (AEM) is an efficient, noncontact geophysical exploration technique that is used primarily to image subsurface conductive structures [1]. It is widely applied in mineral exploration [2], groundwater surveys [3], lithological properties [4] and other fields [5]. However, the inversion of AEM data is limited by the inherent nonlinearity of time-domain electromagnetic responses and the ill-posed nature of the inverse problem [6,7]. Therefore, achieving stable and high-precision inversion of AEM data remains a significant challenge.

Traditional gradient-based inversion algorithms, such as nonlinear conjugate gradient (NLCG) and Gauss–Newton (GN) methods, are widely used optimization techniques to reconstruct subsurface conductivity distributions in geophysical exploration [8,9,10]. However, these algorithms are highly sensitive to the choice of the initial model and are prone to convergence to local minima. Moreover, the objective function usually combines a data misfit term with a model roughness term to stabilize the inversion [10,11], yet such regularization often oversmooths thin layers and anomalous boundaries, thereby resulting in the loss of structural details. On the other hand, global optimization algorithms, such as simulated annealing (SA) and genetic algorithms (GAs), rely heavily on repeated forward modeling, which leads to high computational costs and limits their practicality for complex geoelectrical models [12,13,14].

In recent years, neural networks, with a strong ability to perform nonlinear mapping and feature extraction, have opened up new avenues for geophysical inversion [15,16,17,18,19,20,21,22]. Compared with traditional inversion algorithms, neural networks can efficiently handle high-dimensional data and capture intricate subsurface resistivity structures, thereby significantly improving the resolution and accuracy of the recovered models. However, purely data-driven, end-to-end neural network inversions rely heavily on the representativeness of training datasets and often lack explicit physical constraints or priors, which limits their interpretability and generalizability.

To overcome this limitation, physics-guided learning has attracted increasing attention in geophysical inversion [23,24,25,26,27,28,29]. Existing strategies include adding physics-based loss terms during network training, using forward responses to penalize inconsistent predictions, and providing conventional inversion results as prior information to the network. These approaches improve the physical reliability of neural network inversion, but many of them still operate as one-way prediction frameworks. The network output is not repeatedly corrected by a subsequent physics-based inversion step and therefore lacks a self-correction mechanism when generalization errors occur.

In this paper, we propose a gradient-guided iterative enhancement (GGIE) inversion method for AEM data that is based on GN inversion, U-Net prediction, and an objective-guided adaptive weight method. The main contribution of GGIE is the closed-loop coupling between physics-based correction and learned structural enhancement. The GN module provides a physically constrained inverted model, the U-Net module supplies a structural enhancement proposal, and the adaptive fusion module balances the two according to forward-data consistency [30,31]. The fused model is then used as the starting model for the next GN correction. In this way, the GGIE inversion method does not simply accept the neural network output but repeatedly corrects it with forward physics.

In this paper, the theoretical foundations of the proposed GGIE inversion method, with an emphasis on the GN correction module, the U-Net prediction module, and the adaptive weighted strategy, are first outlined. Synthetic experiments are subsequently conducted using theoretical models to validate the correctness and effectiveness of the GGIE inversion approach. Finally, the algorithm is applied to field AEM data acquired from the Fox River in Wisconsin, USA. By retaining the gradient-based inversion results as physically consistent constraints, adaptively fusing them with the neural network predictions, and returning the fused model to the GN module for correction, the proposed method achieves more accurate and stable inversion of AEM data.

## 2. Methods

### 2.1. Gradient-Guided Iterative Enhancement Inversion

The aim of AEM inversion is to estimate subsurface electrical structure from measured data. We formulate the regularized inversion as the minimization of the following penalty function [32]:

(1) $$\Phi \left(m\right)=\Phi_{d}+\beta \Phi_{m}.$$

where m represents the M-dimensional model vector; $\Phi \left(m\right)$ represents the inversion objective function; $\Phi_{d}$ and $\Phi_{m}$ represent the data-misfit and model-regularization terms, respectively; and β represents the trade-off parameter. The model is updated along a descent direction to search for the solution that minimizes the following objective function:

(2) $$\hat{m}=argmin_{m}\Phi \left(m\right),$$

where $\hat{m}$ denotes the optimal subsurface resistivity model. In conventional gradient-based AEM inversion, the update direction is determined by the gradient of the objective function and the local sensitivity around the current model. This strategy is physically stable, but the recovered model is often affected by the starting model and by regularization-induced smoothing. After one iteration, a coarse inverted model (IM) is obtained using the GN method by solving Equation (2).

Neural network prediction in GGIE inversion can be expressed as follows:

(3) $$PM=N_{\Theta }\left(d^{obs},IM\right),$$

where $N_{\Theta }$ denotes the trained neural network with parameters Θ, $d^{obs}$ represents the observed AEM data, IM represents the current GN-derived inverted model from Equation (2), and PM represents the predicted model provided by the U-Net.

At a given GGIE iteration, the data misfits of the IM and the PM are denoted as $e_{IM}$ and $e_{PM}$, respectively. A smaller misfit indicates good agreement with the observed data and, therefore, a larger provisional contribution is assigned to the corresponding model. The provisional PM weight is computed as follows:

(4) $$\lambda^{*}=clip\left(\frac{\gamma /\left(e_{PM}+\epsilon \right)}{1/\left(e_{IM}+\epsilon \right)+\gamma /\left(e_{PM}+\epsilon \right)},\lambda_{min},\lambda_{max}\right),$$

where γ represents the neural network confidence factor, ϵ represents a small positive number for numerical stability, and $\lambda_{min}$ and $\lambda_{max}$ represent the lower and upper bounds, respectively, of the PM weight. To avoid abrupt oscillation of the weighted coefficient, the provisional weight is further smoothed by a momentum term:

(5) $$\Delta \lambda =\left(\lambda^{*}-\lambda_{prev}\right)+\mu \Delta \lambda_{prev},$$

(6) $$\lambda =clip\left(\lambda_{prev}+\Delta \lambda ,\lambda_{min},\lambda_{max}\right),$$

where μ is the momentum coefficient, $\lambda_{prev}$ is the fusion weight from the previous fusion step, and $\Delta \lambda_{prev}$ represents the previous weight increment. For the first fusion step, no previous fusion weight exists; thus, λ is initialized directly from $\lambda^{*}$. In this study, $\lambda_{min}=0.05$, $\lambda_{max}=0.75$, and μ=0.25. The update model (UM) is constructed in log-resistivity space:

(7) $$UM=\exp\left[\left(1-\lambda \right)\log\left(IM\right)+\lambda \log\left(PM\right)\right].$$

The adaptive fusion weight is calculated from the data relative misfits of the GN-derived model and the U-Net-predicted model. The model with a smaller misfit contributes more to the fused update. To avoid unstable jumps, the U-Net weight is limited to a bounded range and then smoothed by a momentum term. In this study, the U-Net weight is constrained to 0.05–0.75, and the momentum coefficient is set to 0.25. These parameter settings are kept fixed in all synthetic and field-data experiments.

**Figure 1:**
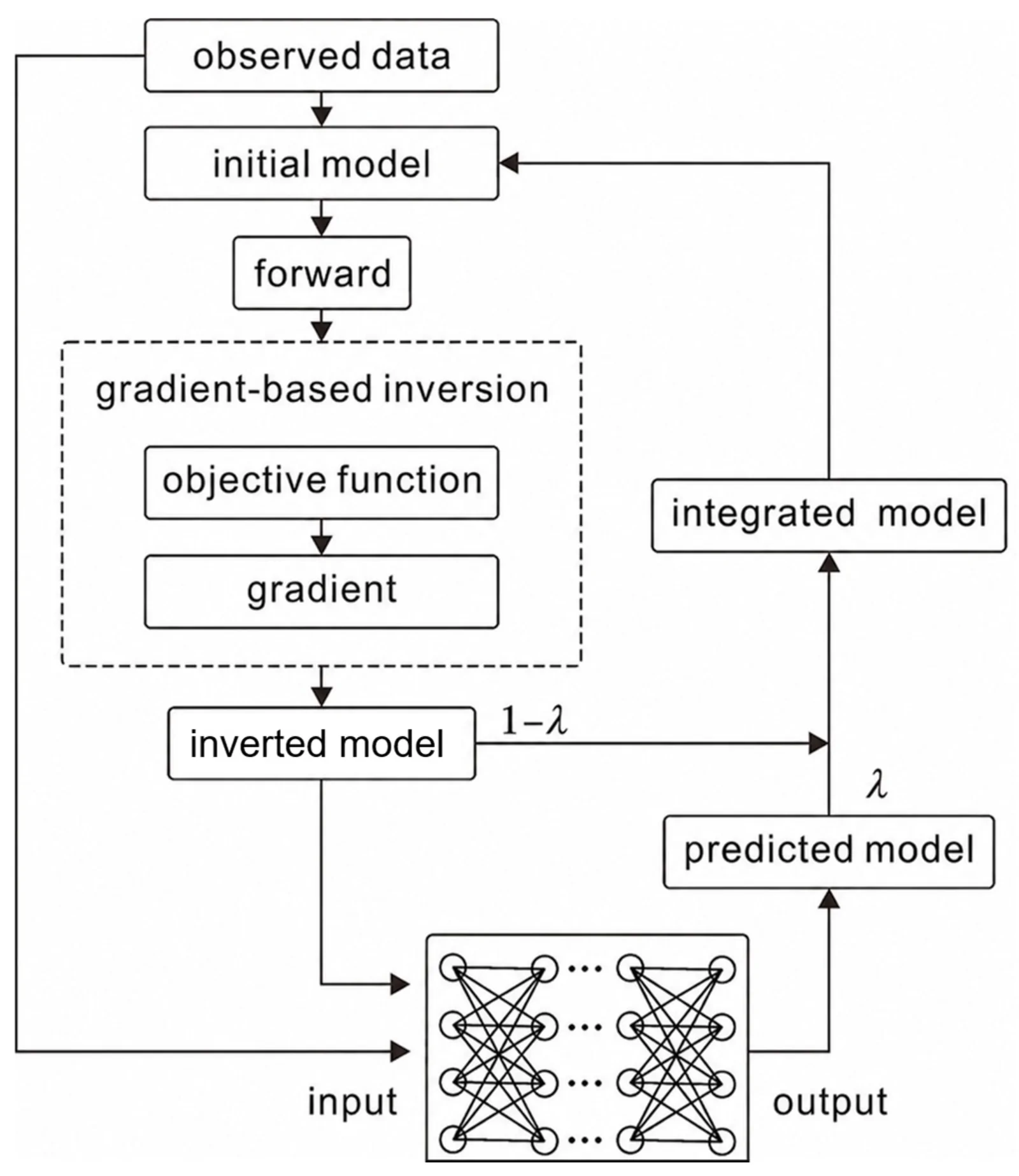
Workflow of GGIE inversion. The algorithm consists of a gradient-based inversion module, a neural network module, and a hybrid model module. Solid arrows indicate the flow of data and models, and the dashed box encloses the gradient-based inversion module. IM, PM, and UM denote the inverted, predicted, and updated models, respectively, while λ and 1−λ denote the corresponding fusion weights.

In this study, according to our sensitivity tests, the maximum number of GGIE iterations is set to 10. The iteration stops earlier if the mean relative error between the AEM data predicted from the current GN-updated model and the observed AEM data falls below the convergence threshold of 10^−3^. The complete GGIE procedure, which is shown in Figure 1, can be summarized as follows.

(1) Gradient-guided module: A low-resolution IM is obtained by performing gradient-based inversion to minimize a regularized objective function, with the model ensured to satisfy Maxwell’s equations.
(2) Enhanced structure module: A PM is output by a neural network with a dual-channel input structure that combines the observed data and the IM to achieve rapid nonlinear mapping and resistivity prediction.
(3) Hybrid model module: A UM is hybridized by balancing the contributions from the IM and the PM (Table 1) through weighting the parameters from Equation (6). This step progressively approaches the true geological distribution over successive iterations.
(4) The UM serves as the initial model for the next round of gradient-based inversion.
(5) By cyclically executing steps (1)–(4), closed-loop physics-constrained and data-driven feedback is established, thereby increasing both the resolution and convergence speed while preserving physical plausibility.

### 2.2. Gradient-Based Inversion

In the GGIE inversion algorithm, the physics-based inversion module is implemented using the open-source SimPEG framework (version 0.24.0; Python version 3.10.19) [33]. The GN inversion minimizes the regularized weighted least-squares objective from Equation (2):

(8) $${\hat{m}}_{\left(GN\right)}=argmin_{m}\left[\Phi_{d}\left(F\left(m\right),d^{obs}\right)+\beta \Phi_{m}\left(m,m_{ref}\right)\right].$$

In this study, the data-misfit and model-regularization terms are defined as follows:

(9) $$\Phi_{d}={\left\|W_{d}\left[d^{obs}-F\left(m\right)\right]\right\|}_{2}^{2},$$

(10) $$\Phi_{m}=\alpha_{s}{\left\|m-m_{ref}\right\|}_{2}^{2}+\alpha_{x}{\left\|D_{z}\left(m-m_{ref}\right)\right\|}_{2}^{2},$$

where $d^{obs}$ represents the observed AEM data, $F\left(m\right)$ represents the one-dimensional layered-earth forward operator, $W_{d}$ represents the data-weighting matrix, $D_{z}$ represents the first-order vertical difference operator, $m_{ref}$ represents the reference model, and β represents the trade-off parameter, which is initially set to 10^3^ and is then progressively reduced during inversion by multiplying it by a cooling coefficient (typically 0.1) as the number of GGIE iterations increases.

In the implementation, the corresponding regularization weights are set to $\alpha_{s}=0.01$ and $\alpha_{x}=1.0$. The data-weighting matrix is defined as $W_{d}=diag\left(1/\epsilon_{i}\right)$, where $\epsilon_{i}=\max\left(0.05\left|d_{i}^{obs}\right|,10^{-15}\right)$. This weighting corresponds to a 5% relative data uncertainty and prevents numerical instability when late-time response amplitudes are very small. In each GGIE iteration, the GN module is run for one iteration step to generate a physically corrected IM that can be used in the next neural network prediction.

### 2.3. Network Architecture and Parameters

Within the GGIE inversion algorithm, the neural network module is responsible for feature extraction from the physics-constrained gradient-based inversion results. This module uses an improved, lightweight U-Net architecture [34] designed to efficiently learn the complex nonlinear mapping relationship between obtained AEM data and subsurface resistivity models.

**Figure 2:**
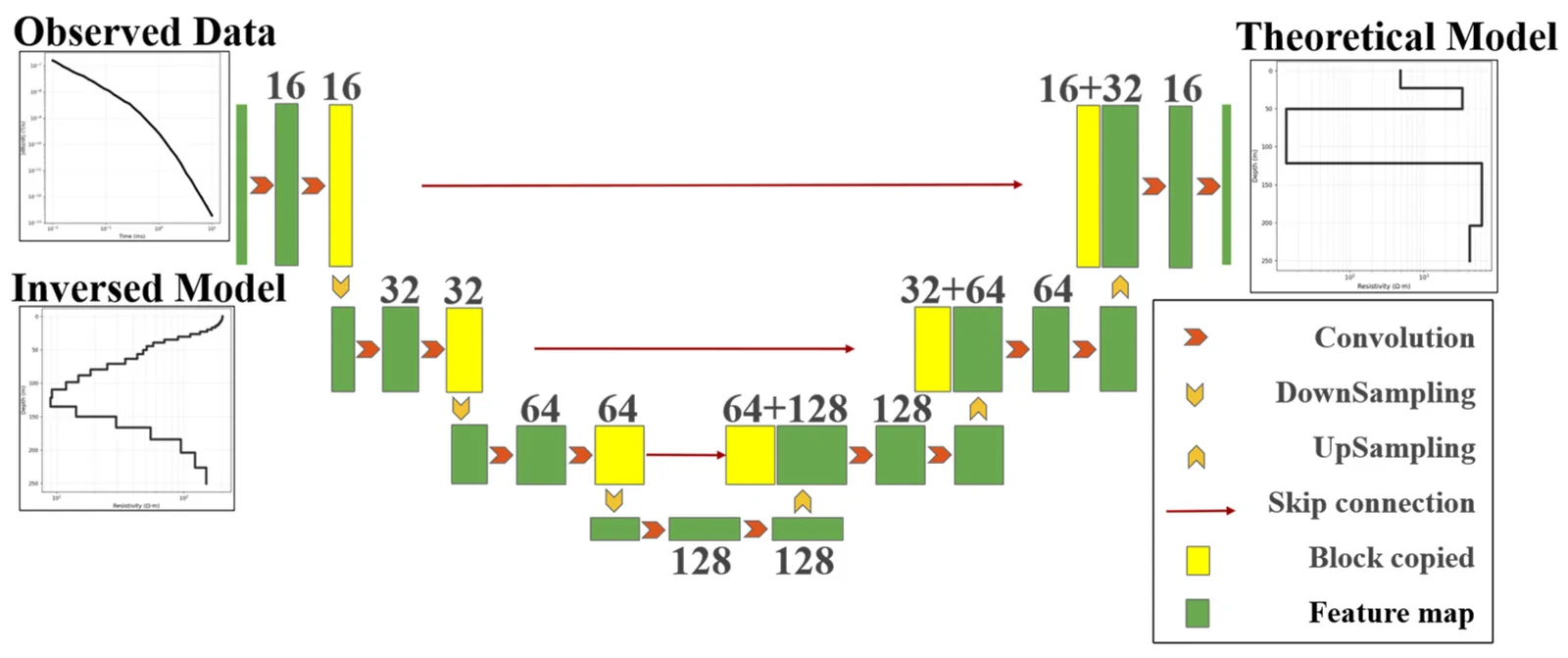
Schematic diagram of the U-Net neural network architecture within GGIE inversion. The network adopts an encoder–decoder architecture that contains multiple layers of convolution, pooling, upsampling, and skip connections to realize high-resolution nonlinear prediction of resistivity models.

The structure of the neural network module within the GGIE inversion algorithm is shown in Figure 2. The network adopts an encoder–decoder structure with skip connections. We introduce visual geometry group convolutional blocks (VGG-Blocks) at each stage of both the encoder and the decoder [35]. Each block contains two consecutive convolution sequences (where each sequence is convolution → batch normalization → LeakyReLU) with a kernel size of 3. This stacked dual-convolution design substantially enlarges the effective receptive field of the network and increases its capacity for nonlinear feature extraction, thereby enabling it to more effectively capture subtle variations in geoelectrical models. During encoding, max pooling (stride = 2) is applied three times for downsampling, thus progressively compressing the spatial dimensions, to extract multiscale abstract features. The decoder uses upsampling to gradually restore spatial resolution. Skip connections directly concatenate high-resolution, shallow features from the encoder into the corresponding decoder stages. This multiscale feature fusion mechanism effectively counteracts detail loss in deeper layers, thereby significantly improving the accuracy of the inverted model in resolving thin layers and stratigraphic interfaces.

The U-Net input is designed as a dual-channel structure that aligns with the GGIE workflow: Channel 1 is the coarse resistivity model derived from the physics-constrained gradient inversion, and Channel 2 receives the observed AEM data.

The network follows a symmetrical encoder–decoder structure with 3 downsampling stages in the encoder, a bottleneck layer, and 3 corresponding upsampling stages in the decoder. The number of feature channels progressively doubles at each downsampling step (16, 32, 64, and 128) before being symmetrically reduced during upsampling and culminating in a single-channel output that represents the predicted 1D resistivity distribution. The configuration of each layer—including the input dimensions, convolutional kernel specifications, and computational cost—is summarized in detail in Table 2.

Although a VGG-style dual-convolution block is used to increase the nonlinear representational capacity of the network, the total number of learnable parameters remains limited to approximately 0.16 M, with a computational cost of approximately 2.47 million floating-point operations (FLOPs). Compared with large-scale vision models, which often contain tens or hundreds of millions of parameters, the proposed architecture retains a highly lightweight footprint. This “compact yet expressive” design ensures adequate feature-learning ability while substantially reducing the computational and memory overhead, thereby enabling efficient integration into the rapid closed-loop iteration of the GGIE framework and meeting the practical requirements for real-time AEM inversion.

### 2.4. Dataset Construction

**Figure 3:**
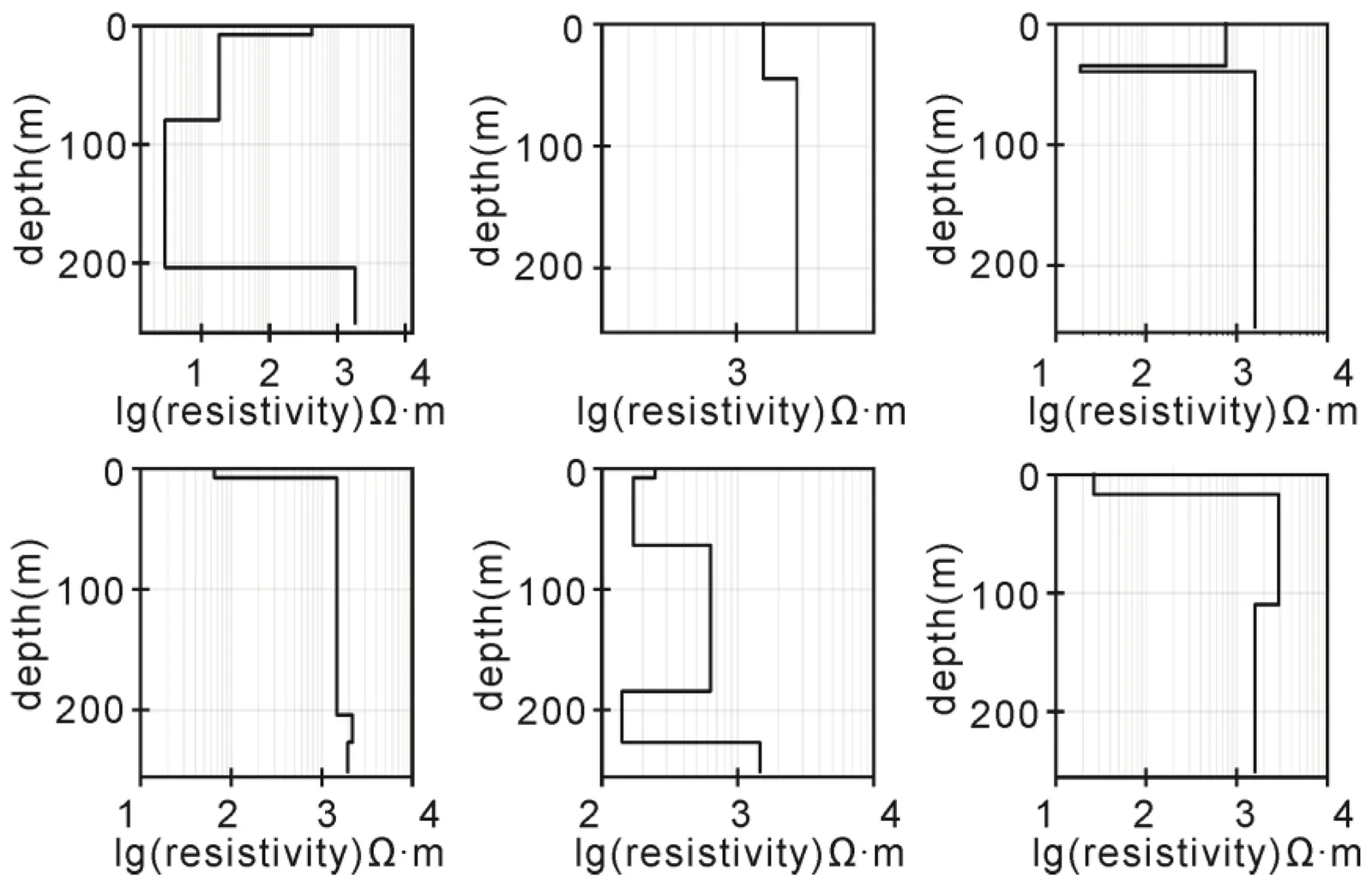
Examples of typical one-dimensional resistivity geoelectric models randomly generated for the training dataset. These models are generated by randomizing parameters to simulate diverse subsurface geological conditions, thereby improving the generalizability of the neural network and its ability to capture complex structures.

On the basis of the differences in the conductivity of subsurface media, a total of 10,000 theoretical models with 2 to 6 layers were randomly generated to cover different typical geological structures (Figure 3). The model library covers H-, A-, K-, and Q-type layered models as well as more complex multilayer structures, thus providing different interface combinations and resistivity contrasts. During model generation, layer resistivities and thicknesses were randomly sampled in logarithmic space to cover a wide range of geological scenarios, from high-resistivity bedrock to low-resistivity ore bodies, to ensure that the dataset reflects the diversity of actual subsurface structures. After forward modeling, relative Gaussian noise levels of 3%, 5%, and 10% were added to the synthetic AEM data as noise-augmented samples so that the U-Net module learns not only from ideal noise-free responses. The dataset was then randomly partitioned into a training set (8000 samples), a validation set (1000 samples), and a test set (1000 samples) in a ratio of 8:1:1.

Recent AEM studies have increasingly combined numerical forward modeling, synthetic data generation, and data-driven inversion to improve electromagnetic imaging efficiency and model recovery [36,37,38]. To ensure that the synthetic data align with actual surveys, forward modeling of the AEM was performed using the typical acquisition parameters of the United States Geological Survey (USGS) SkyTEM 2022 survey (SkyTEM304M system).

### 2.5. Data Normalization and Network Training

Because both AEM data and resistivity values span several orders of magnitude, logarithmic normalization was applied before network training as follows:

(11) $$x_{norm}=\frac{\mathrm{lg}\left|x\right|-\mathrm{lg}x_{min}}{\mathrm{lg}x_{max}-\mathrm{lg}x_{min}}.$$

where x denotes the raw input or output variable and $x_{min}$ and $x_{max}$ represent the minimum and maximum values, respectively, in the corresponding dataset. This normalization scales the data to a comparable numerical range and prevents large-amplitude channels from dominating the training process.

**Figure 4:**
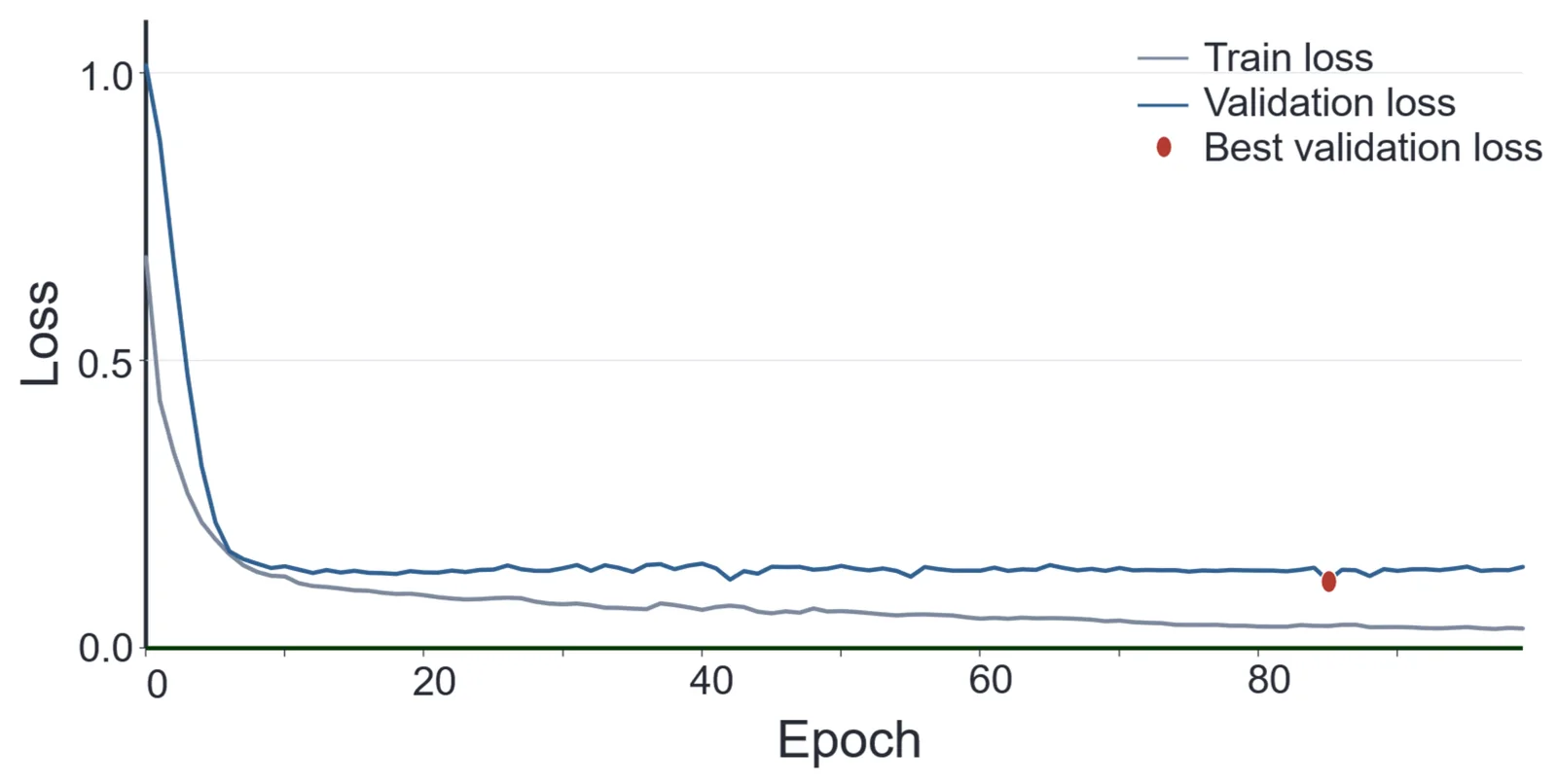
Training and validation loss curves of the U-Net neural network.

The U-Net is trained once before the GGIE loop and is kept fixed during iterative inversion. During GGIE inversion, the network is used only for prediction; no additional network training is performed inside the loop. The network is trained using the mean squared error (MSE) between the normalized predicted model and the normalized target model. The training and validation loss curves are shown in Figure 4. Because this loss has the same squared-error form as the root mean square (RMS)-type evaluation metrics defined in Section 2.6, its formula is not repeated here. The network parameters are optimized using the Adam optimizer in PyTorch (version 2.9.1; CUDA version 12.8). A dynamic learning-rate strategy is used: a higher learning rate accelerates early convergence, whereas a lower learning rate improves later-stage fine-tuning.

### 2.6. Evaluation Metrics

To evaluate the inversion performance from complementary perspectives, three quantitative metrics are used: model relative error, log-domain RMSE, and RMS relative data misfit. The first two metrics are used evaluate the model-domain recovery accuracy, whereas the third metric is used evaluate the data-domain physical consistency.

The relative error of the model is defined as follows:

(12) $$E_{m}=\frac{1}{L}\sum_{l=1}^{L}\left|\frac{\rho_{l}^{pred}-\rho_{l}^{true}}{\rho_{l}^{true}}\right|\times 100\%,$$

where L represents the number of model layers, $\rho_{l}^{pred}$ represents the recovered resistivity of the l-th layer, and $\rho_{l}^{true}$ represents the corresponding target resistivity.

The log-domain RMSE and RMS relative data misfit are both RMS-type metrics and can be written in the following unified form:

(13) $$R=\sqrt{\frac{1}{N}\sum_{i=1}^{N}{\left[\frac{z_{i}^{pred}-z_{i}^{ref}}{s_{i}}\right]}^{2}}.$$

For log-domain RMSE, $z_{i}^{pred}=\log_{10}\left(\rho_{i}^{pred}\right)$, $z_{i}^{ref}=\log_{10}\left(\rho_{i}^{true}\right)$, $s_{i}=1$, and N=L. This metric is used to evaluate the model recovery over a wide resistivity range. For the RMS relative misfit, $z_{i}^{pred}=d_{i}^{pred}$, $z_{i}^{ref}=d_{i}^{obs}$, $s_{i}=d_{i}^{obs}$, and N=T, where T represents the number of time gates. This metric is used to evaluate whether the recovered model is physically consistent with the observed AEM data. The model relative error and log-domain RMSE are used to evaluate the model-domain accuracy, whereas the RMS relative misfit is used to evaluate the data-domain physical consistency.

## 3. Robustness and Generalization Analysis

### 3.1. Iterative Enhancement and Stability

**Figure 5:**
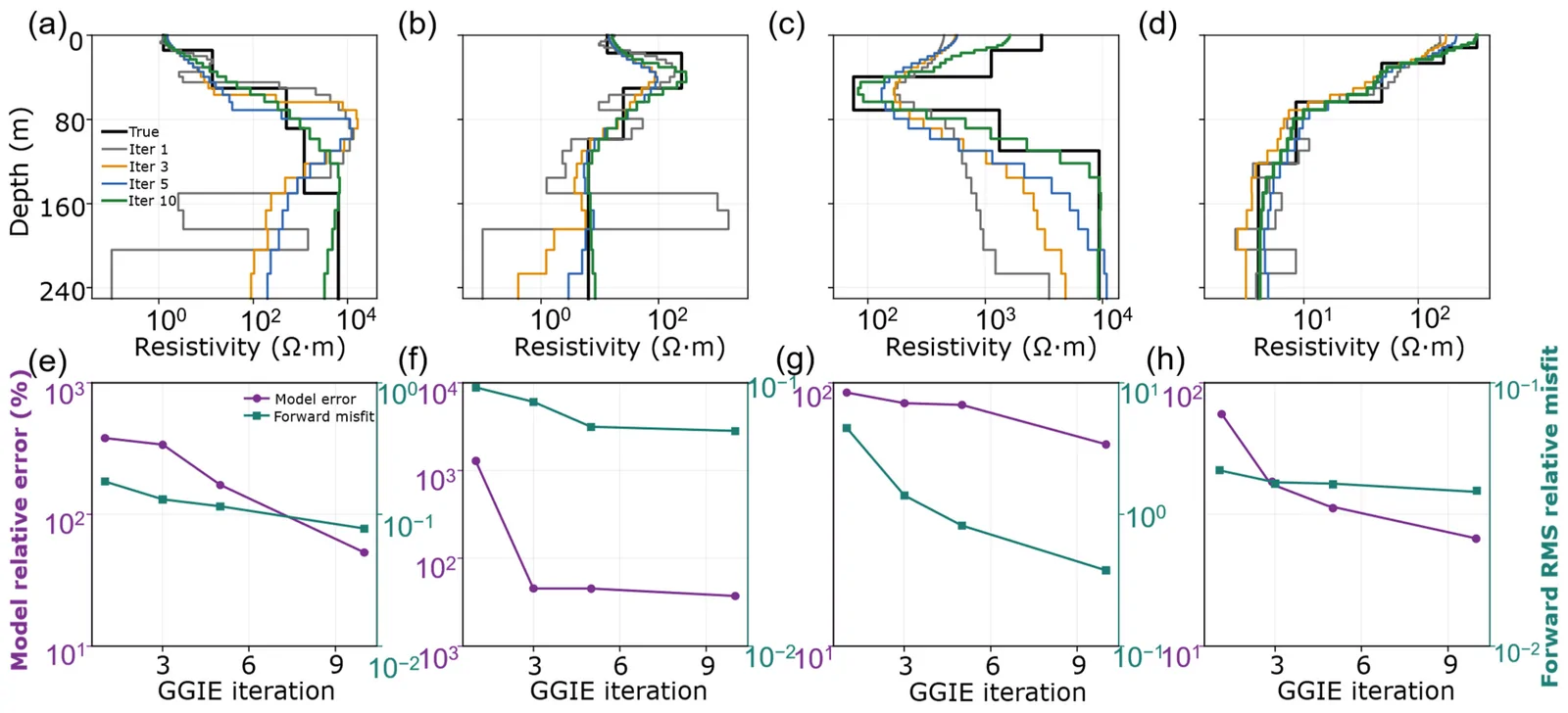
Iterative profiles and convergence curves of GGIE for representative H-, A-, K-, and Q-type theoretical models. (**a**–**d**) Resistivity profiles at iterations 1, 3, 5, and 10. The black line represents the true model. (**e**–**h**) Corresponding evolution of the relative error of the model and the forward RMS relative misfit. The results show that GGIE progressively improves the recovered model and maintains data-domain consistency within the selected iteration range.

To examine the iterative behavior of GGIE inversion, the model evolution and error variation are shown in Figure 5. The early-iteration results still deviate from the true models, especially near thin layers and sharp resistivity transitions. With increasing iteration number, the GGIE profiles gradually approach the true models, and the main interfaces become more accurately positioned. Both the relative error of the model and the RMS relative misfit decrease over the selected iterations (Figure 5e–h). This finding indicates that the iterative process improves the recovered model in the model domain while maintaining consistency with the observed AEM response in the data domain.

**Figure 6:**
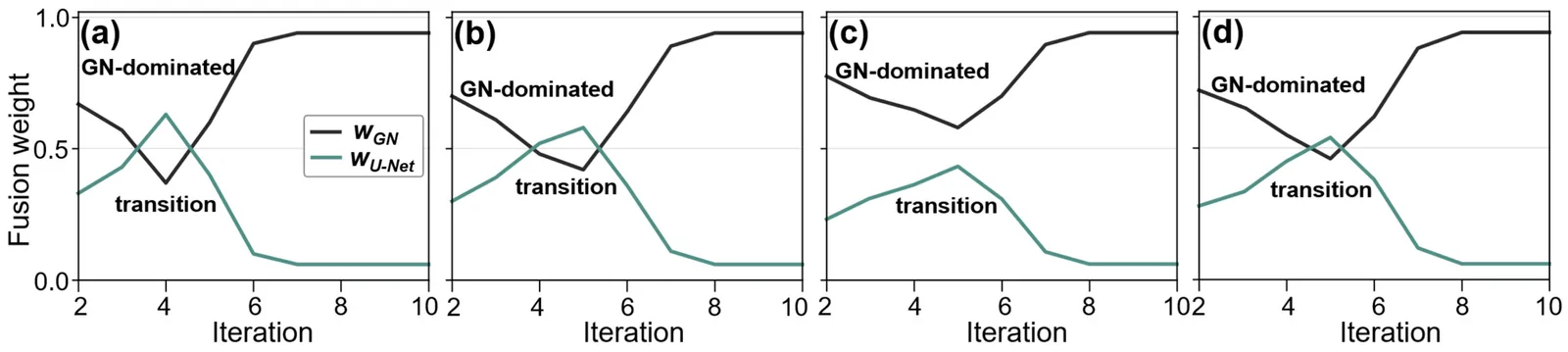
Adaptive fusion-weight evolution for representative H-, A-, K-, and Q-type theoretical models. (**a**–**d**) Fusion weights for the four model types. The black line denotes the GN-derived IM weight, and the green line denotes the U-Net-predicted PM weight. The curves show a GN-dominated early stage, a transition stage with increased U-Net contribution, and a stable bounded stage after approximately the eighth iteration.

The adaptive fusion weight evolution for the representative H-, A-, K-, and Q-type models is shown in Figure 6. The weight curves reveal three stages during the GGIE iteration process: model convergence, structural enhancement, and data fitting. In the early stage, the GN-derived IM weight is relatively high because the IM is obtained by minimizing the regularized objective function and therefore retains stronger physical consistency. In the middle stage, the U-Net-predicted PM weight increases and usually reaches its maximum near the fifth iteration, which indicates that the learned structural enhancement contributes more strongly to the fused model at this transition stage.

In the late stage, the IM weight increases again and approaches the imposed upper bound. This increase occurs because the initial beta ratio used for GN correction decreases by one order of magnitude in each GGIE iteration. According to the current implementation, the initial beta ratio is $10^{3}$ at the first iteration and decreases to $10^{-1}$ at the fifth iteration. This progressive decrease weakens the influence of model regularization and makes the GN correction more data fitting oriented. By approximately the eighth iteration, the fusion weights reach a stable bounded state, which indicates that the inversion has entered a physically consistent data-fitting stage while retaining the structural information introduced by the U-Net. This behavior indicates that the main correction is achieved within the 10-iteration limit.

### 3.2. Robustness to Noise

**Figure 7:**
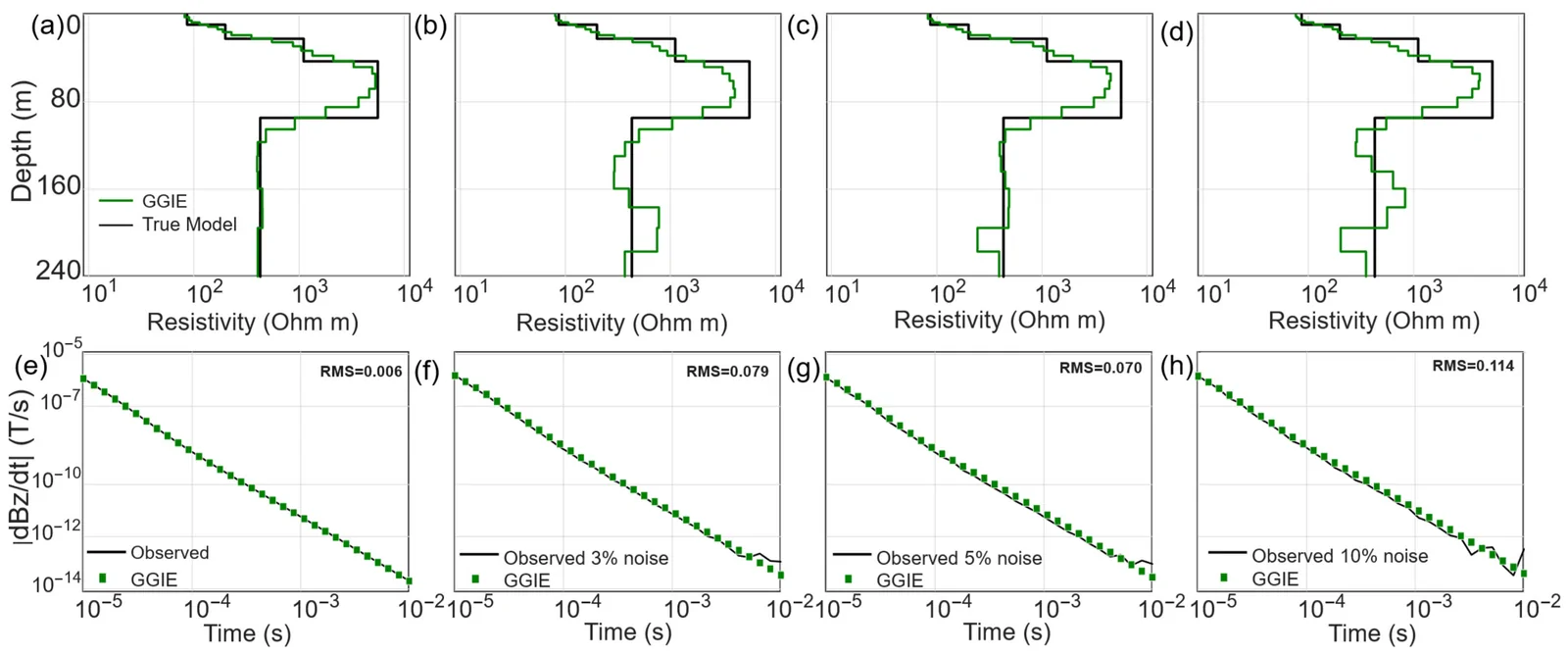
Noise robustness test of GGIE on an additional complex multilayer model: (**a**–**d**) recovered resistivity profiles for noise-free AEM data and data with 3%, 5%, and 10% noise, respectively, compared with the true model; (**e**–**h**) the corresponding observed and GGIE-predicted responses. The RMS relative misfit is specified in each data-fitting panel.

To further test the robustness of GGIE to noise, an additional complex multilayer model is generated and inverted under noise-free and 3%, 5%, and 10% noisy AEM data. As shown in Figure 7, the recovered profiles still follow the main resistivity trend of the true model under the different noise levels. The data-fitting curves also remain close to the observed responses. The RMS relative misfits are 0.006, 0.079, 0.070, and 0.114 for the noise-free and 3%, 5%, and 10% noise cases, respectively. The 10% noise case shows a larger deviation than the lower-noise cases do, but the main layered structure is still retained. These results indicate that the noise-augmented training set, together with the GN-guided closed-loop correction, improves the robustness of GGIE under moderate noise perturbations.

### 3.3. Sensitivity to Training Dataset Size

To evaluate the sensitivity of GGIE to the amount of training data, nested synthetic training libraries containing 2000, 5000, and 10,000 samples were constructed. The network architecture, training settings, AEM configuration, and GGIE inversion parameters were kept unchanged. All three models were evaluated on the same 100 independent test models.

**Figure 8:**
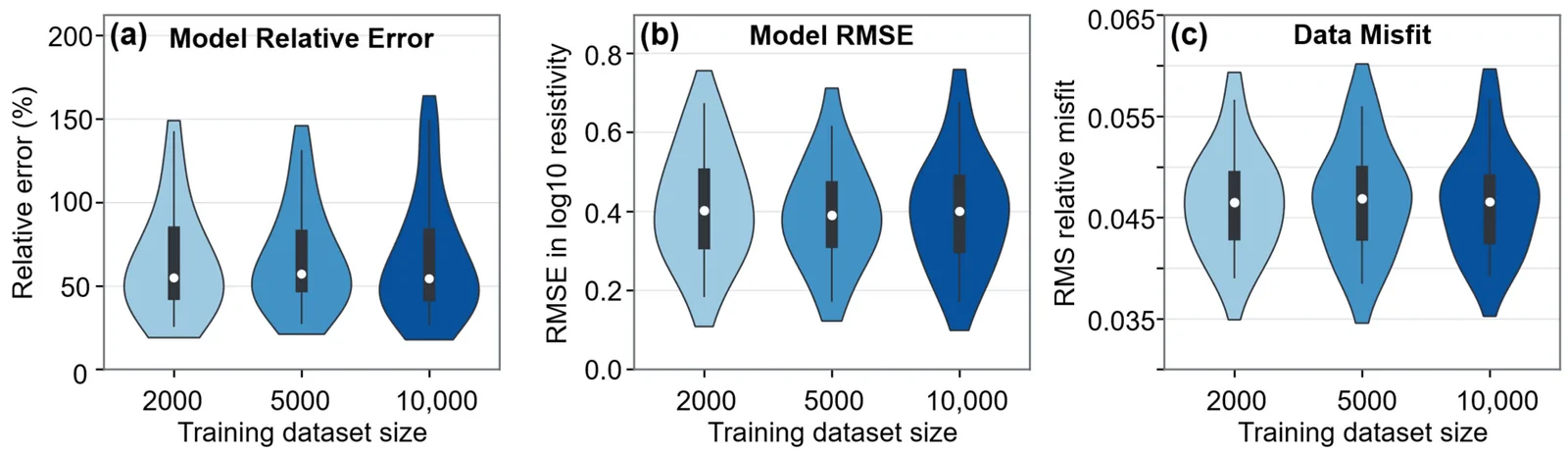
Violin plots showing the distributions of (**a**) model relative error, (**b**) log-domain RMSE, and (**c**) RMS relative data misfit for GGIE trained on 2000, 5000, and 10,000 samples over the same 100 independent test models.

As shown in Figure 8 and Table 3, the model relative errors obtained using 2000, 5000, and 10,000 training samples are 55 ± 22%, 57 ± 26%, and 56 ± 25%, respectively. The corresponding log-domain model RMSE values are 0.42 ± 0.15, 0.40 ± 0.14, and 0.40 ± 0.15, while the RMS relative data misfits are 0.0531 ± 0.0096, 0.0502 ± 0.0080, and 0.0506 ± 0.0084. The strongly overlapping distributions and small differences among these metrics indicate that GGIE maintains stable inversion performance across the tested training-set sizes [39].

For GGIE, the same uniform 100 Ωm half-space is used to initialize both the three-layer and complex multilayer tests. Despite their different structural complexities, GGIE recovers the main resistivity trends without model-specific initial information because each fused prediction is subsequently corrected by GN inversion. This demonstrates robustness to the initial model and supports adaptability across the tested geological structures. Together with the noise-robustness results in Section 3.2, these findings show that the GN-guided closed-loop correction reduces the sensitivity of GGIE to the amount of training data within the tested range.

## 4. Synthetic Data Experiments

### 4.1. Simple Synthetic Model

**Figure 9:**
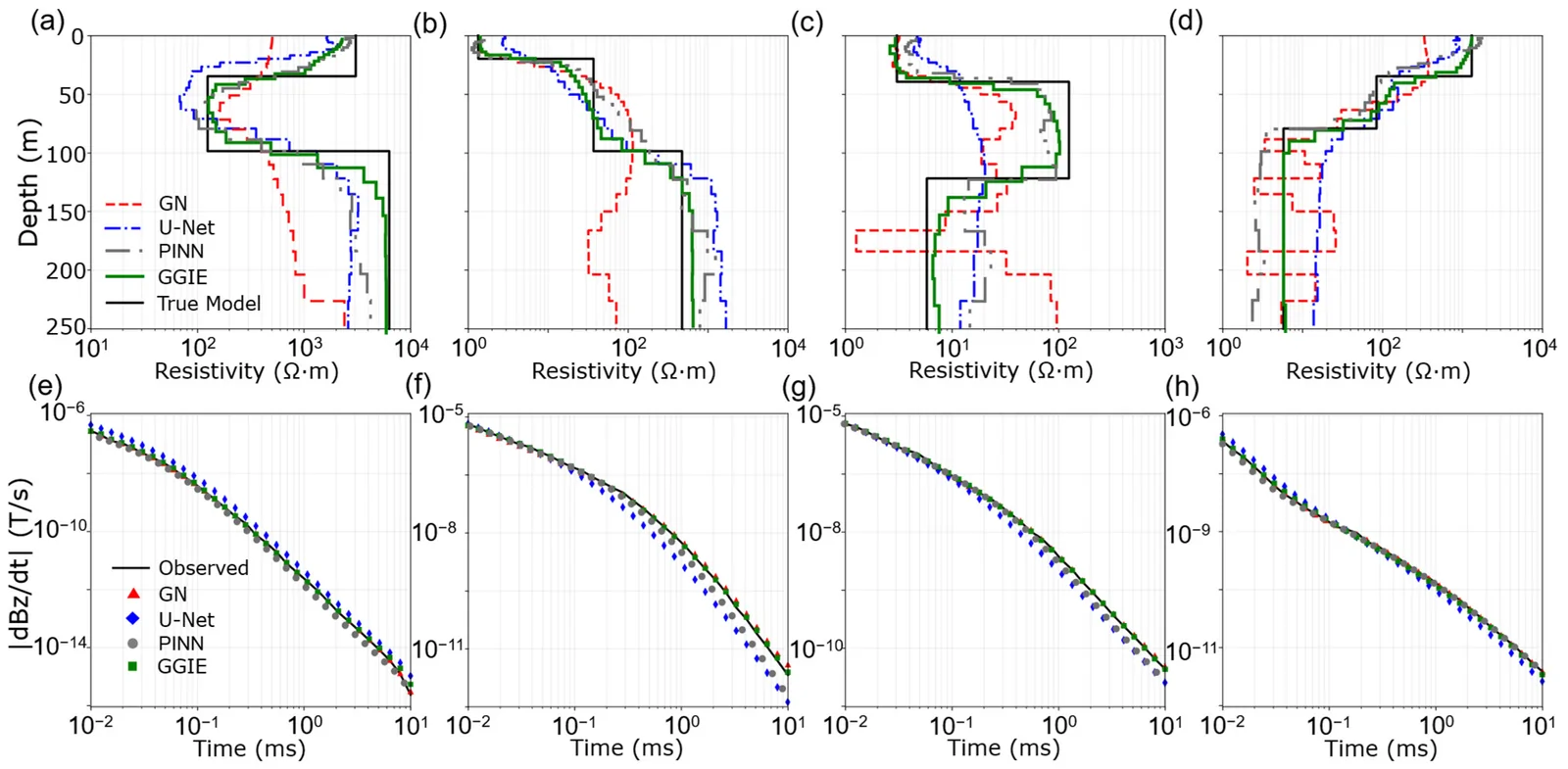
Comparison of inversion results for typical three-layer geoelectric models. (**a**–**d**) Recovered resistivity profiles for the H-, A-, K-, and Q-type models. The red dashed, blue dash-dotted, gray dash-dotted, green solid, and black solid lines denote GN, U-Net, PINN, GGIE, and the true model, respectively. (**e**–**h**) Corresponding data-fitting curves.

To evaluate the basic behavior of the proposed method, four representative three-layer geoelectric models, including H-, A-, K-, and Q-type structures, were selected. These models represent common basement-cover configurations and provide a relatively low-dimensional test for comparing conventional GN inversion, U-Net-only prediction, PINN prediction, and the proposed GGIE inversion [25,40]. The recovered resistivity profiles and corresponding data fits are shown in Figure 9.

For the simple three-layer models shown in Figure 9, all four methods recover the main geoelectric trend. GN-only inversion recovers a smooth resistivity model but has difficulty resolving the middle or deep resistive layers. This limitation results mainly from the regularization term in Equation (1). U-Net-only prediction produces sharper interfaces, but it may introduce oscillations or deviations that are not supported by the forward response. The PINN adds forward-response consistency during training, which reduces some U-Net deviations and improves physical consistency. Nevertheless, because the PINN produces a direct prediction without iterative physics-based correction during inference, local errors remain in interface depth and resistivity magnitude.

In contrast, GGIE inversion integrates the stability of GN correction with the feature-extraction ability of the network in a closed loop. Across the four profiles, GGIE recovers the layer boundaries and resistivity values more consistently than the one-pass U-Net and PINN predictions. The corresponding data-fitting curves are shown in Figure 9e–h. The GGIE responses remain closely aligned with the synthetic observations, confirming that structural enhancement is achieved without sacrificing data consistency.

### 4.2. Complex Multilayer Model

**Figure 10:**
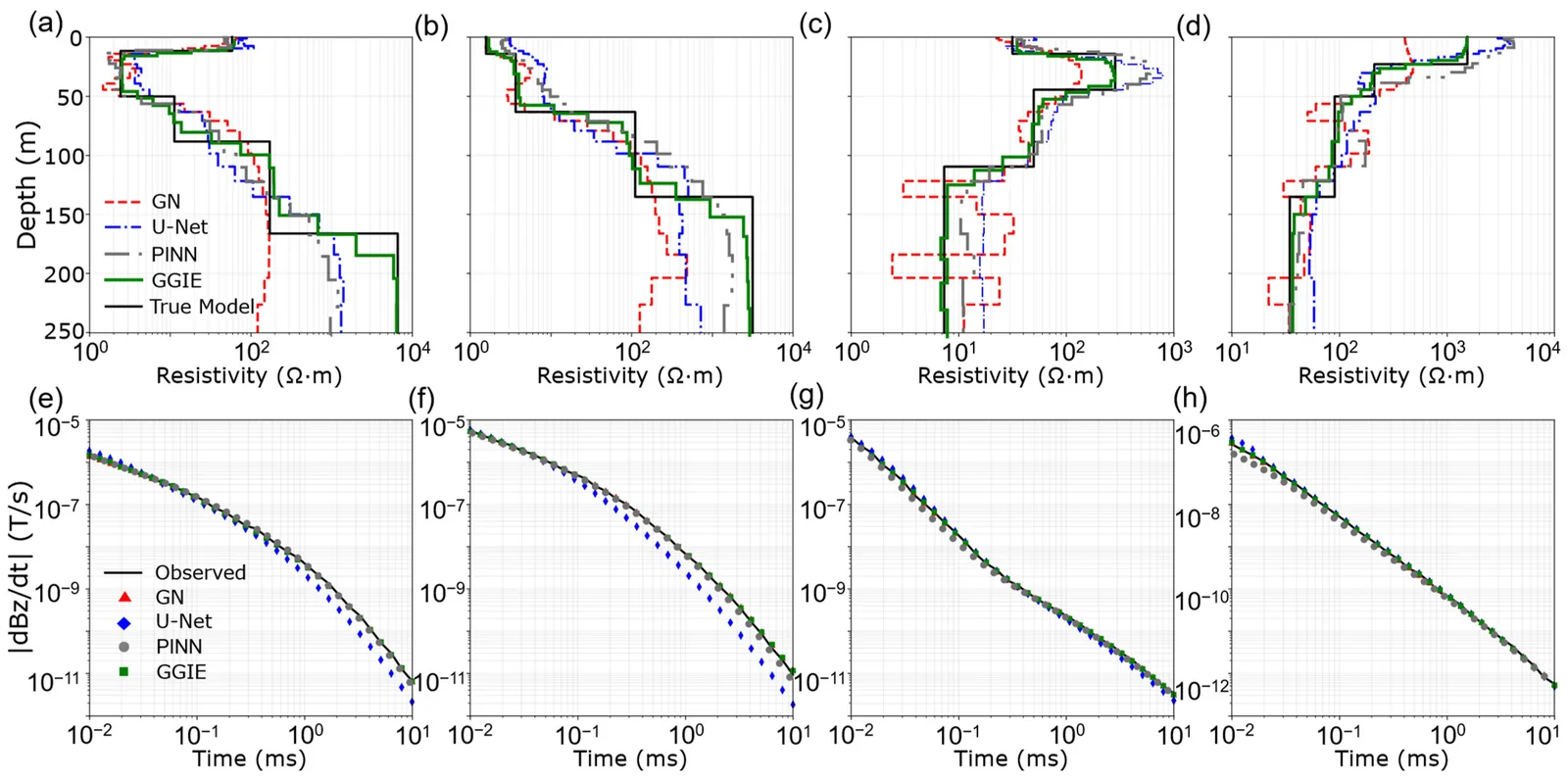
Comparison of inversion results for complex five-layer geoelectric models. (**a**–**d**) Recovered resistivity profiles for models containing alternating resistivity contrasts and thin interbeds. The red dashed, blue dash-dotted, gray dash-dotted, green solid, and black solid lines denote GN, U-Net, PINN, GGIE, and the true model, respectively. (**e**–**h**) Corresponding data-fitting curves.

The advantage of GGIE becomes more evident for the complex five-layer models shown in Figure 10. Compared with the three-layer examples in Figure 9, these models contain more interfaces, alternating high- and low-resistivity layers, and thinner interbeds. Such structures are more challenging for AEM inversion.

The results in Figure 10a–d show that GN inversion recovers the broad resistivity trend but smooths thin layers and sharp boundaries. This limitation is especially visible for the thin conductive layer in Figure 10a and the thin resistive layer in Figure 10c. U-Net-only prediction produces sharper variations but may shift interfaces or introduce extra oscillations. PINN suppresses some data-inconsistent variations through its forward-response loss, although several thin layers remain smoothed or displaced because it performs a single-pass, non-iterative inversion at inference time.

GGIE inversion reduces these limitations by combining learned structural enhancement with iterative GN correction. In Figure 10a, GGIE preserves the conductive interbed rather than smoothing it into the surrounding layers. In Figure 10c, GGIE also provides a clearer expression of the thin resistive layer. Compared with PINN, the repeated GN correction moves the enhanced model toward both the target structure and the observed response. Consequently, the GGIE profiles follow the target models more closely in thin-layer regions.

The AEM data-misfit curves in Figure 10e–h show that the responses predicted from the GGIE models agree well with the observed synthetic responses. GGIE inversion is used to balance structural resolution and data fitting.

### 4.3. Error Analysis

**Figure 11:**
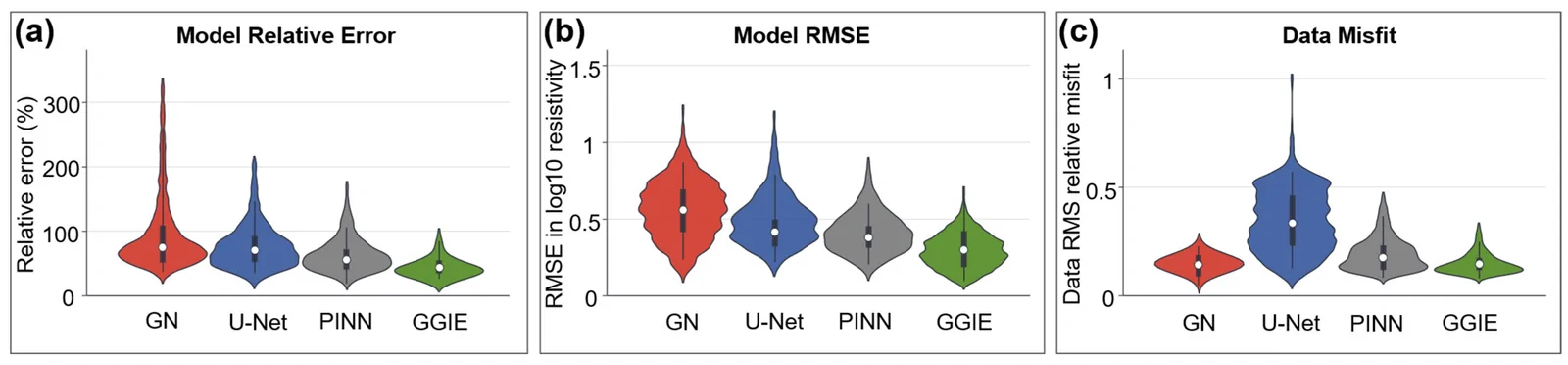
Violin plots showing the distributions of (**a**) model relative error, (**b**) log-domain RMSE, and (**c**) RMS relative data misfit for GN, U-Net, PINN, and GGIE over 1000 test samples.

The model relative error, log-domain RMSE, and RMS relative data misfit for each method are shown in Figure 11 and reveal that GGIE inversion achieves the most concentrated error distribution with the smallest variance. A statistical analysis on the basis of 1000 test samples is illustrated in Table 4. GGIE inversion achieves the lowest model relative error of 50 ± 17%, which is approximately 49.0%, 34.2%, and 19.4% lower than those of GN inversion (98 ± 67%), U-Net-only prediction (76 ± 34%), and PINN prediction (62 ± 26%), respectively. It also has the lowest log-domain RMSE of 0.40 ± 0.14, which is approximately 28.6%, 9.1%, and 7.0% lower than those of GN inversion (0.56 ± 0.20), U-Net-only prediction (0.44 ± 0.17), and PINN prediction (0.43 ± 0.16), respectively. Furthermore, GGIE inversion maintains the lowest RMS relative data misfit of 0.0508 ± 0.0037, which is comparable to that of GN inversion (0.0510 ± 0.0041) and substantially lower than those of U-Net-only prediction (0.3482 ± 0.1491) and PINN prediction (0.1793 ± 0.0869), corresponding to reductions of 85.4% and 71.7%, respectively. These results demonstrate the advantage of GGIE inversion in improving inversion accuracy while maintaining physical consistency with the observed AEM data.

By retaining the gradient-based inversion result as a physically consistent constraint and simultaneously leveraging the strong nonlinear mapping ability of the neural network to provide structurally enhanced model updates, the proposed method achieves more accurate and stable inversion of AEM data.

## 5. Field Data Experiment

**Figure 12:**
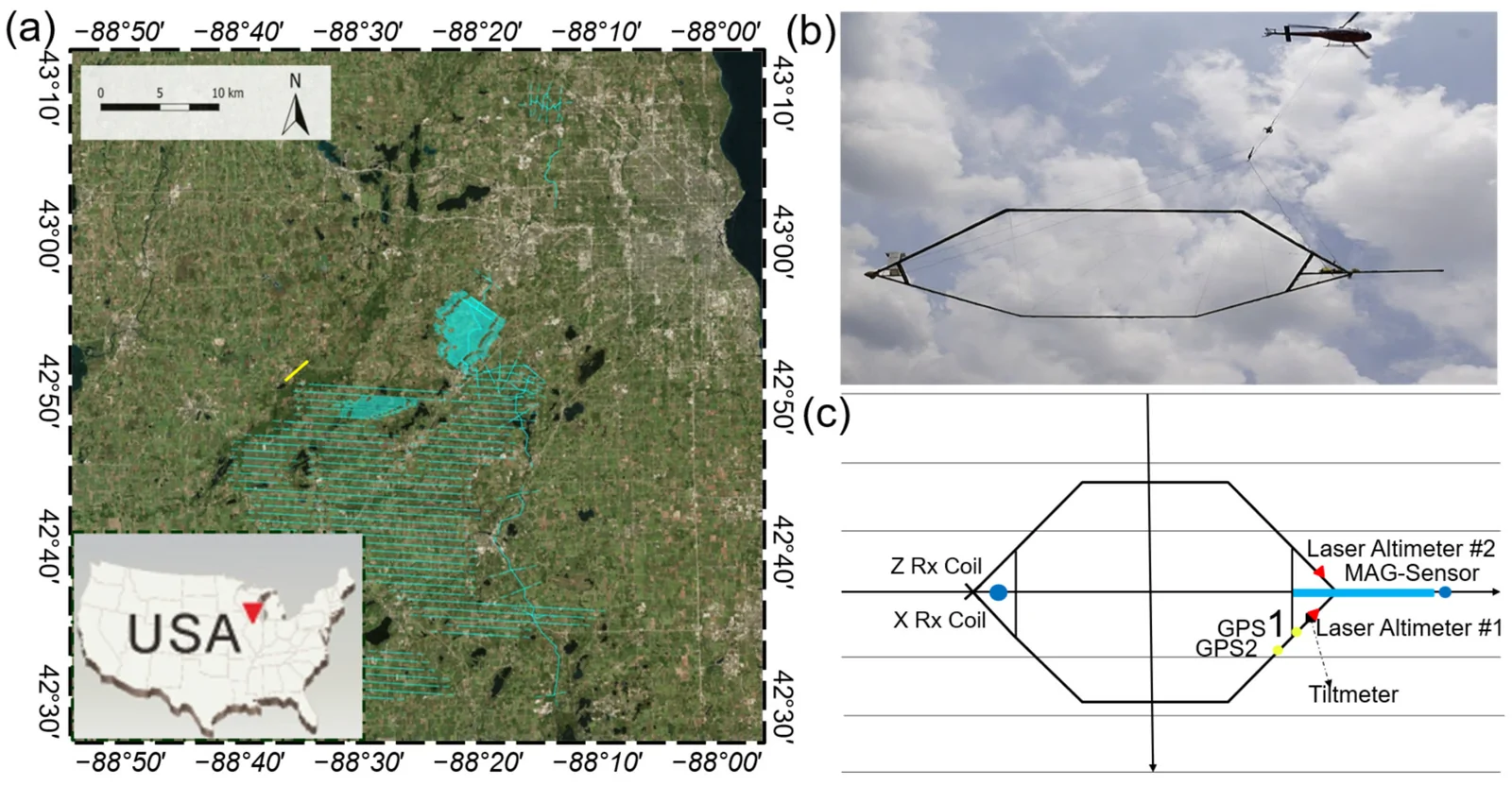
Location map of the field survey area (**a**) and the configuration (**b**) and acquisition geometry (**c**) of the SkyTEM304M AEM system. The red triangle denotes the location of the survey area in Wisconsin, USA. The main map displays satellite imagery overlaid with the spatial distribution of the SkyTEM flight lines (cyan lines). The yellow solid line (L516201) is selected for GGIE inversion.

The field AEM data were collected by the SkyTEM304M system (SkyTEM Surveys ApS, Aarhus N, Denmark) in March 2022 [41]. The survey area was located in the Fox River region of Wisconsin, USA (Figure 12). These data are used to refine hydrogeologic units for groundwater models and to improve the accuracy for geologic formations. The geographical locations and distribution of the flight lines overlaid on the satellite imagery are shown in Figure 12a. The configuration and acquisition geometry of the SkyTEM304M AEM system are shown in Figure 12b,c, respectively. The transmitter loop is configured as a rigid hexagon with an area of 342 m^2^ and is positioned approximately 30 m above the ground. The time channels consist of 32 time gates covering the standard SkyTEM sampling interval from $10^{-5}$ to $10^{-2}$ s, with a receiver Z-coil effective area of 105 $m^{2}$. To demonstrate the ability of GGIE to enhance field data, we selected Flight Line 516201 (highlighted in yellow in Figure 12a) for detailed inversion analysis.

### 5.1. Representative Field Soundings

**Figure 13:**
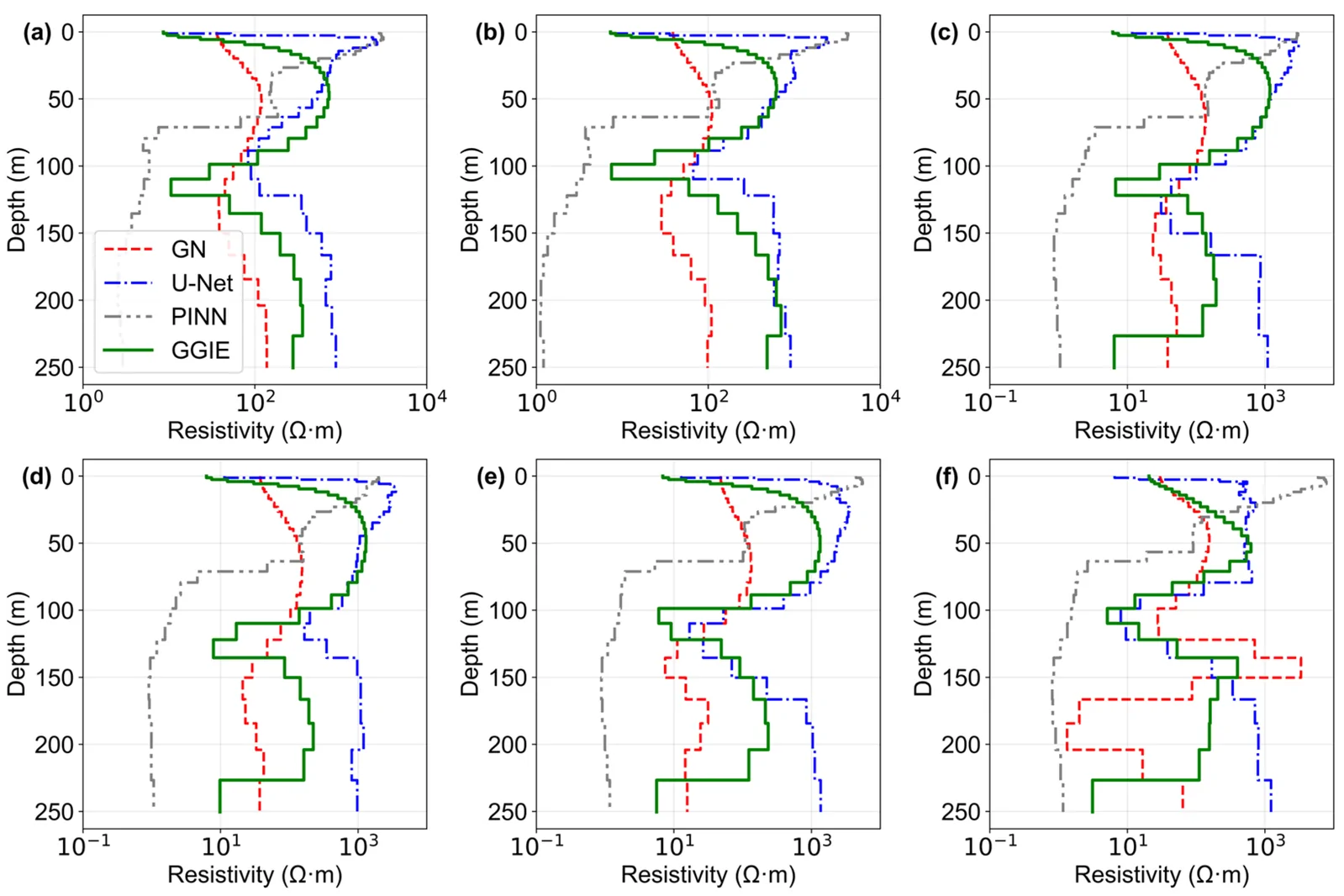
Comparison of inversion results for six representative field soundings (**a**–**f**). The red dashed, blue dash-dotted, gray dash-dotted, and green solid lines denote GN, U-Net, PINN, and GGIE, respectively.

The inversion results for six points along Flight Line 516201 are compared in Figure 13. The model recovered using only GN inversion is smooth. The U-Net-only prediction provides sharper resistivity variations. The PINN prediction incorporates forward-response consistency. However, as a single-pass, non-iterative inversion at inference time, it still exhibits local resistivity deviations at several soundings. In contrast, GGIE inversion preserves the structural enhancement provided by the network while correcting the updated model through the GN module. Therefore, compared with the profiles obtained by U-Net and PINN, the GGIE inversion profiles show more continuous resistivity transitions and improved physical consistency.

### 5.2. Data Fitting

**Figure 14:**
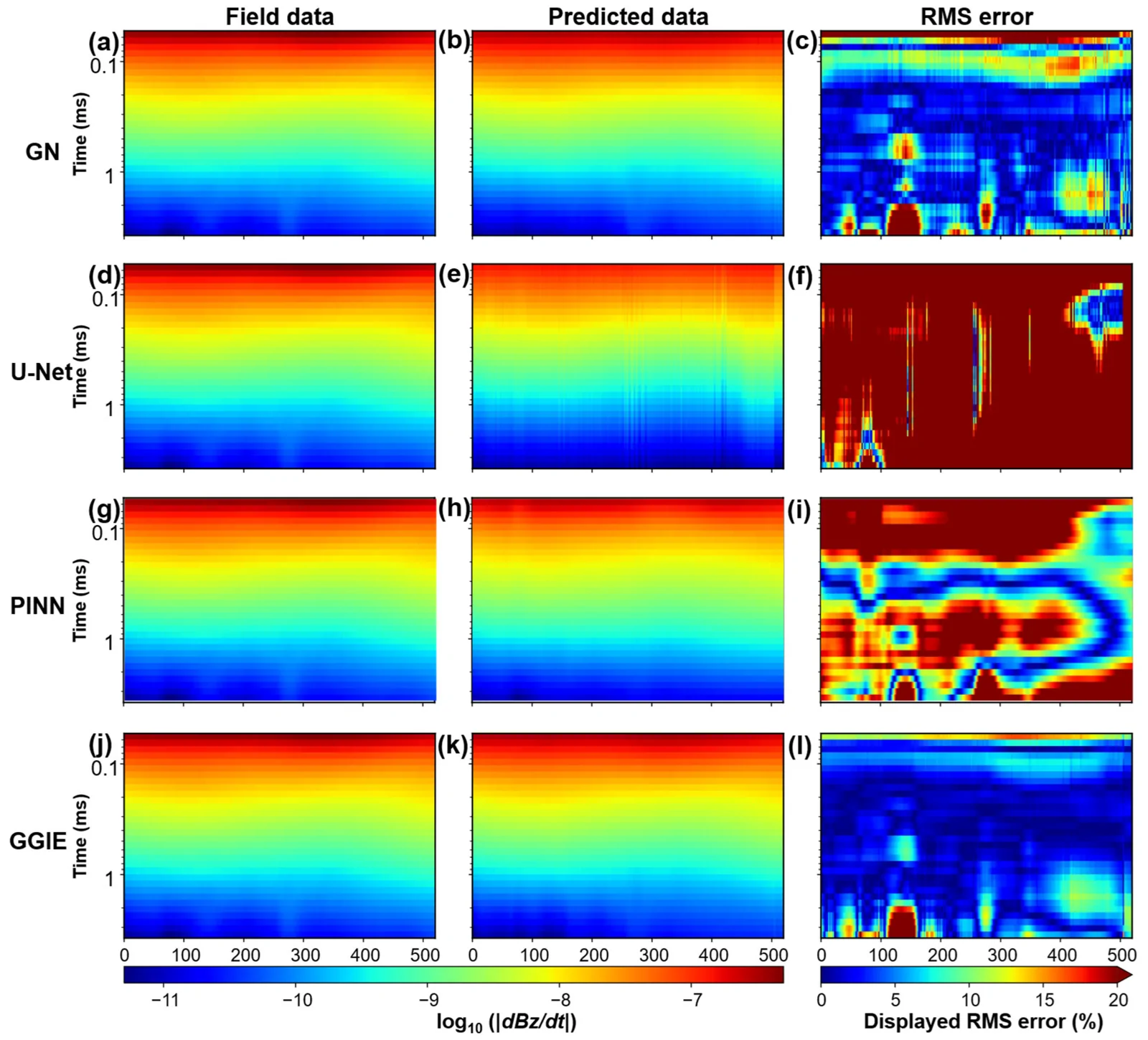
Comparison of observed and predicted field AEM data for GN, U-Net, PINN, and GGIE. The first, second, and third columns show the field data, predicted data, and displayed RMS error, respectively. Rows (**a**–**c**), (**d**–**f**), (**g**–**i**), and (**j**–**l**) correspond to GN, U-Net, PINN, and GGIE, respectively.

The data misfits and errors are shown in Figure 14. GN inversion provides a more stable data fit because each model update is guided by the forward response calculated from the current model and by the data-misfit term between the predicted and observed AEM data. However, localized high-error zones remain along the line (Figure 14a–c). The U-Net-only prediction shows widespread high-error regions (Figure 14d–f), indicating that a purely data-driven prediction can generate resistivity structures whose forward responses deviate substantially from the field data. The PINN prediction reduces some of these widespread errors by incorporating forward-response consistency during training, although several coherent high-error regions remain (Figure 14g–i). The GGIE inversion result has a lower and more spatially continuous error distribution, with most residuals concentrated in limited areas rather than distributed across the whole section (Figure 14j–l).

**Figure 15:**
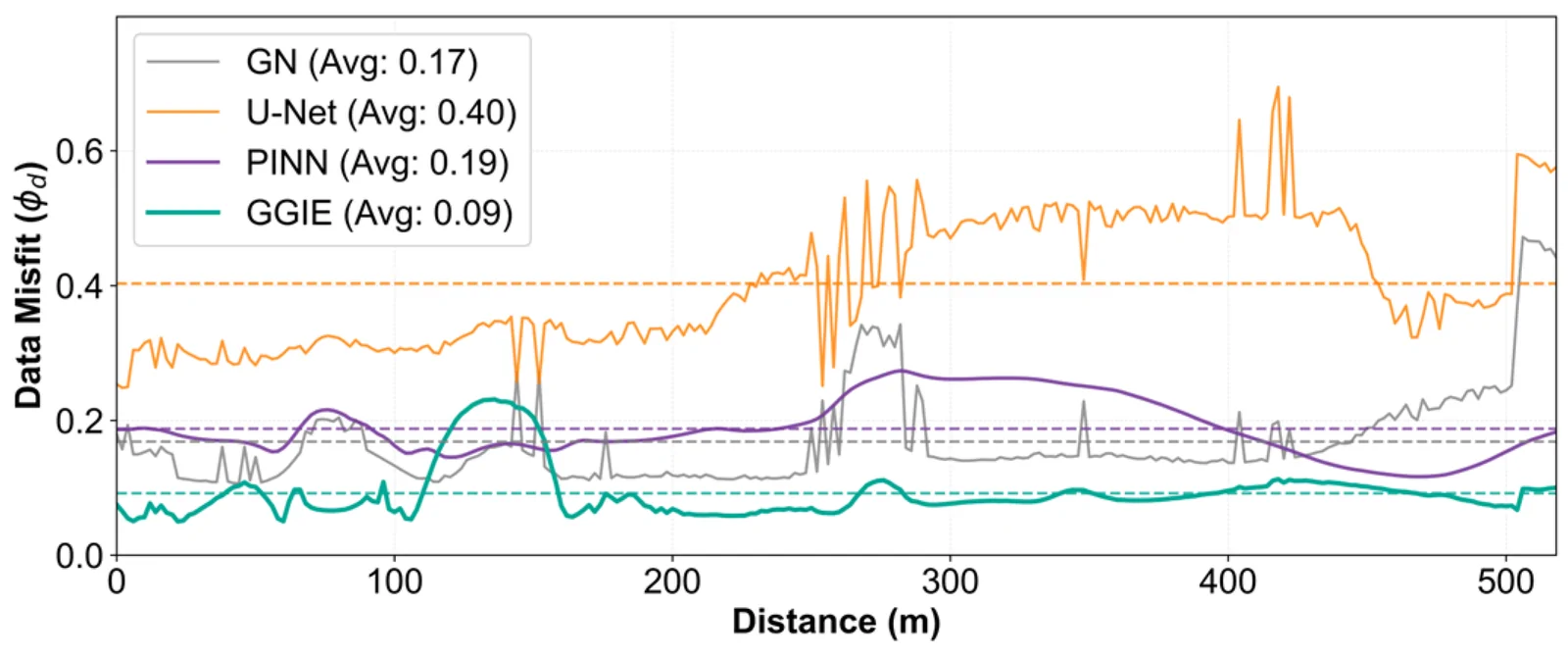
RMS relative data misfit along Flight Line 516201 for GN, U-Net, PINN, and GGIE. Dashed horizontal lines indicate the average misfit of each method. GGIE has the lowest average misfit and shows more stable data fitting along the flight line.

The RMS relative misfit along the full flight line is summarized in Figure 15. The U-Net-only prediction curve is the largest and most unstable, with an average misfit of 0.40. This finding confirms that network prediction alone is insufficient to guarantee data-domain consistency in field data. The PINN prediction reduces the average misfit to 0.19 by incorporating forward-response consistency during training, but its misfit remains higher than those of GN and GGIE. GN inversion reduces the average misfit to 0.17, but several local peaks remain. GGIE inversion achieves the lowest average misfit of 0.09 and maintains a more stable misfit level over most of the profile. This improvement over GN, U-Net, and PINN demonstrates that the closed-loop GGIE strategy does not merely sharpen the inverted image; it also improves the consistency between the recovered model and the observed AEM data.

### 5.3. Inversion Results

**Figure 16:**
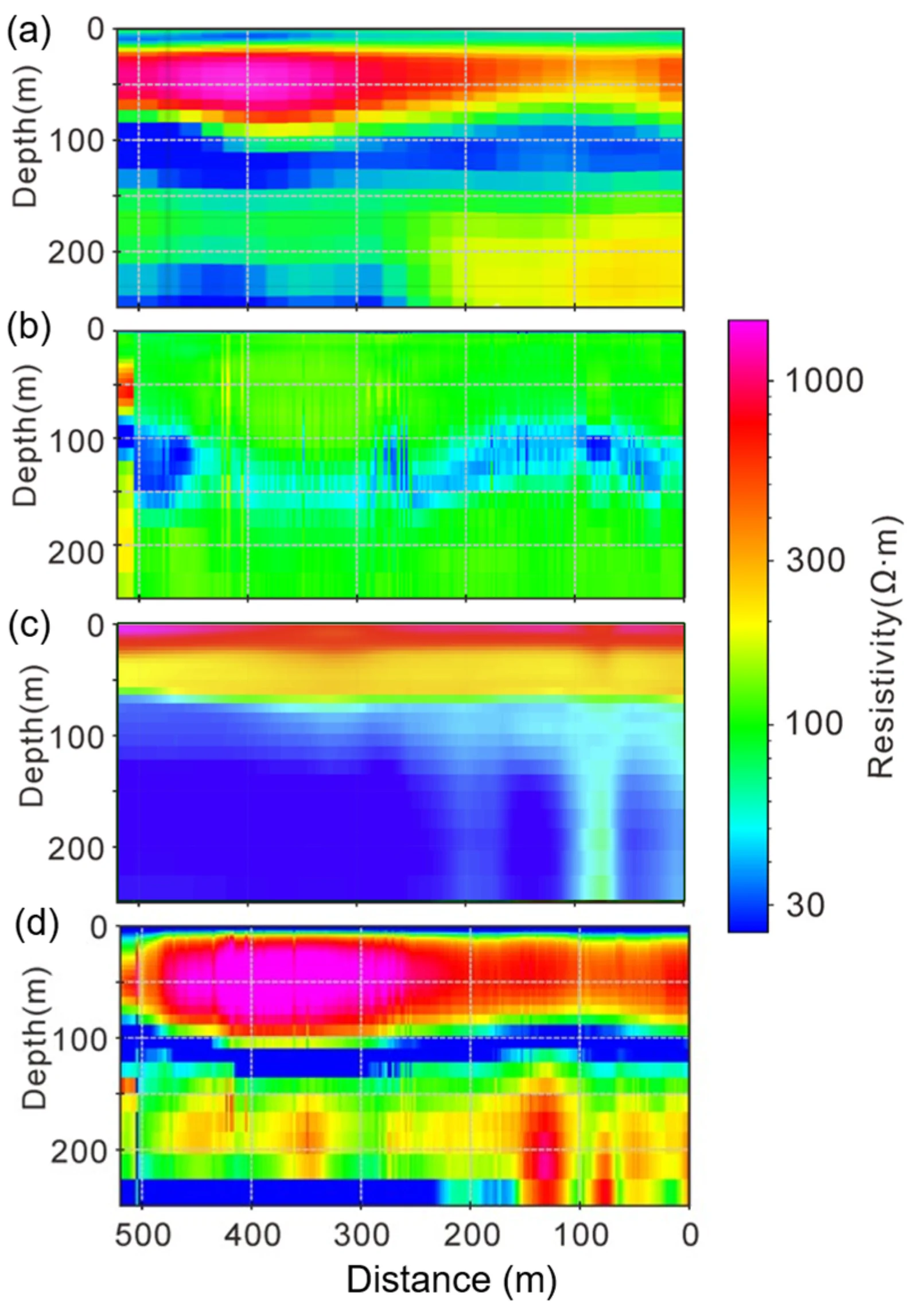
Comparison of resistivity inversion profiles for Flight Line 516201. (**a**) Traditional SCI result from the survey report. (**b**) U-Net-only prediction, which approximately recovers the macroscopic high- to low- to high-resistivity framework but contains discontinuous local anomalies. (**c**) PINN prediction. (**d**) GGIE inversion result. A comparison with the U-Net-only and PINN results reveals that GGIE improves the continuity and physical consistency of the field inversion profile by correcting the learned structural tendency through GN inversion.

The traditional inversion profile from the survey report, which was obtained using the spatially constrained inversion (SCI) algorithm implemented in Aarhus Workbench, is shown in Figure 16a. Because these results are obtained from the USGS Fox River survey report, they are used in this study as the main reference for checking the depth variation in layer boundaries and the lateral continuity of thin resistive and conductive layers. The SCI result can also be regarded as a conventional gradient-based inversion reference.

These results reflect the macroscopic high–low–high geoelectric trend, but the profile is affected by regularization-induced smoothing and therefore has limited interface resolution. The U-Net-only prediction result is shown in Figure 16b. The neural network can only approximately recover the high–low–high resistivity framework: a relatively resistive shallow part, a conductive middle zone, and a more resistive deeper background. However, the predicted profile contains discontinuous band-like features and local unstable anomalies, indicating that the network prediction alone does not provide a sufficiently reliable field inversion result. The PINN prediction result is shown in Figure 16c. By incorporating forward-response consistency during training, PINN reduces some data-inconsistent variations compared with U-Net-only prediction. However, because PINN performs a single-pass, non-iterative inversion at inference time, its profile still shows excessive smoothing and local deviations in interface depth. The GGIE inversion result is shown in Figure 16d. The main interfaces in the GGIE section are highly consistent with the SCI depth trend, and the thin conductive or resistive intervals can still be followed laterally along adjacent soundings.

By combining the structural tendency learned by U-Net with GN-based physical correction, GGIE inversion produces a more continuous and geologically interpretable profile while maintaining improved data-domain consistency.

## 6. Discussion

Within the GGIE inversion algorithm, the U-Net can be viewed as a learned model resolution operator rather than as a standalone inverse solver. The network receives the observed AEM data and the current GN-derived model and outputs a structurally enhanced prediction. Unlike a classical model-resolution matrix, this learned operator is not fixed, linear, or explicitly constructed from the sensitivity matrix. Instead, it is data conditioned and model dependent: its effective resolving kernels vary with the observed response, the current inversion state, and the statistical structure learned from the synthetic training models. Therefore, compared with a conventional model-resolution matrix, the term learned model resolution operator more accurately describes the role of the neural network in GGIE.

This interpretation also clarifies the division of work between the neural-network module and the GN module. The U-Net supplies a resolution-enhancement proposal by reintroducing layer-boundary and thin-layer information that may be weakened by smooth regularization. The subsequent GN inversion is then tested and corrected through the electromagnetic forward operator. Thus, the network acts as a data-conditioned resolving-kernel operator that improves structural separability, whereas the GN module enforces physical data consistency. The closed-loop update prevents the learned prior from becoming an unconstrained image-to-model mapping.

The GGIE inversion contains three coupled functions: model convergence, structural enhancement, and data fitting. These functions are not independent. From the beginning of the iteration, the GN module constrains the model through the electromagnetic forward operator, the U-Net module provides learned structural enhancement, and the adaptive fusion coefficient controls the relative contribution of the two modules. The dominant factor changes during iteration, but the three functions always interact with and constrain one another.

## 7. Conclusions

In this study, a gradient-guided iterative enhancement inversion algorithm for time-domain AEM data was developed. The method couples a GN-based physical inversion module with a lightweight U-Net prediction module. A key component of this coupling is the adaptive model-fusion coefficient, which determines the relative contributions of the GN-derived inverted model and the U-Net-predicted model according to their physical consistency. The U-Net provides a learned structural prior from the observed AEM data and the GN-derived coarse model. In this way, GGIE combines learned model-resolution enhancement with physics-based data consistency. The results of synthetic experiments demonstrate that GGIE is particularly effective for complex multilayer resistivity models. Field data experiments reveal that GGIE produces a more continuous and geologically interpretable resistivity profile.

GGIE is a practical strategy for improving time-domain AEM inversion when both structural resolution and electromagnetic data fitting are needed. In future work, we will focus on uncertainty-aware updating and sensitivity analysis of the fusion weights, which will be extended to multiparameter inversion, where the resistivity and polarization-related parameters are updated together under physical constraints. Its performance will also be compared with other deep-learning-based inversion methods.

## Figures and Tables

**Table 1:** Definition and comparison of different resistivity models used in the proposed GGIE algorithm.

Full Name	Symbol	Methodology	Core Algorithm	Characteristics
Inverted Model	IM	Gradient-based Inversion	Gauss–Newton (GN)	Gradient-guided
Predicted Model	PM	Neural Network	U-Net	Enhanced structure
Updated Model	UM	Hybrid Strategy	GGIE	All

**Table 2:** Architecture and main parameters of the U-Net network.

Stage	Data Size/Channels	Convolution Kernel Sizes/Strides	Params	FLOPs (M)
Input	1 × 32/2	-	0	0
Downsampling 1	1 × 32/16	[1 × 3/1] × 2	960	0.06
Downsampling 2	1 × 16/32	[1 × 3/1] × 2	4800	0.15
Downsampling 3	1 × 8/64	[1 × 3/1] × 2	18,816	0.29
Bottleneck	1 × 4/128	[1 × 3/1] × 2	74,496	0.59
Upsampling 1	1 × 8/64	[1 × 3/1] × 2	49,536	0.79
Upsampling 2	1 × 16/32	[1 × 3/1] × 2	12,480	0.39
Upsampling 3	1 × 32/16	[1 × 3/1] × 2	3168	0.2
Final Layer	1 × 32/1	1 × 1/1	17	0.001
Total	-	-	~164,273	~2.47 M

**Table 3:** Performance of GGIE with different training-set sizes.

Training Set Size	Model Relative Error (%)	Model RMSE	Data Misfit
2000	55 ± 22	0.42 ± 0.15	0.0531 ± 0.0096
5000	57 ± 26	0.40 ± 0.14	0.0502 ± 0.0080
10,000	56 ± 25	0.40 ± 0.15	0.0506 ± 0.0084

**Table 4:** Comparison of error metrics among different inversion methods.

Method	Model Relative Error (%)	Model RMSE	Data Misfit
GN	98 ± 67	0.56 ± 0.20	0.0510 ± 0.0041
U-Net	76 ± 34	0.44 ± 0.17	0.3482 ± 0.1491
PINN	62 ± 26	0.43 ± 0.16	0.1793 ± 0.0869
GGIE	50 ± 17	0.40 ± 0.14	0.0508 ± 0.0037

## Data Availability

The field AEM data used in this study are publicly available from the U.S. Geological Survey data release, Airborne Electromagnetic (AEM) Survey in Southwest and Southeast Areas, Wisconsin, 2022, at https://doi.org/10.5066/P90K1CRG. The processed examples and model outputs generated in this study are available from the corresponding author upon reasonable request.
